# Phosphate fertilizer is a main source of arsenic in areas affected with chronic kidney disease of unknown etiology in Sri Lanka

**DOI:** 10.1186/s40064-015-0868-z

**Published:** 2015-02-24

**Authors:** Channa Jayasumana, Saranga Fonseka, Ashvin Fernando, Kumudika Jayalath, Mala Amarasinghe, Sisira Siribaddana, Sarath Gunatilake, Priyani Paranagama

**Affiliations:** Faculty of Medicine & Allied Sciences, Rajarata University of Sri Lanka, Saliyapura, 50008 Sri Lanka; Faculty of Science, University of Kelaniya, Colombo, 11600 Sri Lanka; College of Chemical Sciences, Institute of Chemistry, Rajagiriya, 10100 Sri Lanka; Department of Health Science, California State University Long Beach, Long Beach, CA 90840 USA

**Keywords:** Arsenic, Pesticides, Fertilizer, Chronic kidney disease of unknown etiology, Sri Lanka

## Abstract

Chronic Kidney Disease of unknown etiology (CKDu) has escalated into an epidemic in North Central Province (NCP) and adjacent farming areas in the dry zone of Sri Lanka. Studies have shown that this special type of CKD is a toxic nephropathy and arsenic may play a causative role along with a number of other heavy metals. We investigated the hypothesis that chemical fertilizers and pesticide could be a source of arsenic. 226 samples of Fertilizers and 273 samples of pesticides were collected and analyzed using atomic absorption spectrometry and inductively coupled plasma mass spectrometry for arsenic and other heavy metals in two university laboratories. Almost all the agrochemicals available to the farmers in the study area are contaminated with arsenic. The highest amount was in triple super phosphate (TSP) with a mean value of 31 mg/kg. Also TSP is a rich source of other nephrotoxic metals including Cr, Co, Ni, Pb and V. Annually more than 0.1 million tons of TSP is imported to Sri Lanka containing approximately 2100 kg of arsenic. The next highest concentration was seen in the rock phosphate obtained from an open pit mine in NCP (8.56 mg/kg). Organic fertilizer contained very low amounts of arsenic. Arsenic contamination in pesticides varied from 0.18 mg/kg to 2.53 mg/kg although arsenic containing pesticides are banned in Sri Lanka. Glyphosate the most widely used pesticide in Sri Lanka contains average of 1.9 mg/kg arsenic. Findings suggest that agrochemicals especially phosphate fertilizers are a major source of inorganic arsenic in CKDu endemic areas. Organic fertilizer available in Sri Lanka is comparatively very low in arsenic and hence the farmers in CKDu endemic areas in Sri Lanka should be encouraged to minimize the use of imported chemical fertilizer and use organic fertilizers instead.

## Background

During the last 2600 years, farmers inhabiting dry zone of Sri Lanka were cultivating rice using irrigated water and organic fertilizer. In the last two decades, escalating numbers of patients with a chronic kidney disease were reported from rural Sri Lanka especially from the North Central Province (NCP) (Jayasumana et al. 2013). Ministry of Health (MoH), Sri Lanka named it as the Chronic Kidney Disease of unknown etiology (CKDu) (Ministry of Health 2009). A WHO led study found the prevalence of CKDu among the 15–70 year olds to be at 15.1% in Anuradhapura and 20.6% in the Polonnaruwa, the two districts of the NCP (Jayatilake et al. 2013).

Patients with CKDu do not have the commonly known risk factors for kidney disease such as diabetes and hypertension (Athuraliya et al. 2011). Histo-pathological findings in the kidneys of CKDu patients include tubular interstitial nephritis associated with mononuclear cell infiltration, glomerular sclerosis and tubular atrophy (Nanayakkara et al. 2012a). This picture of tubulo-interstitial disease with negative immunofluorescence for IgG, IgM, and complement are in favor of toxic nephropathy (Athuraliya et al. 2011). This disease is characterized clinically by tubular proteinuria; alpha-1 and beta-2 microglobulinuria (Nanayakkara et al. 2012b). The observed geographical distribution of the disease and associated socioeconomic characteristics are suggestive of an environmental and occupational etiology. Several studies have been conducted to determine the cause of CKDu, and five such studies had speculated about the causative role of agrochemicals (Peiris-John et al. 2006; Bandara et al. 2010; Wanigasuriya et al. 2011; Jayasumana et al. 2013; Jayatilake et al. 2013). Two of the current authors have formulated a detailed hypothesis that incriminates glyphosate, arsenic and heavy metal complexes as a causative factor for CKDu among paddy farmers in rural Sri Lanka (Jayasumana et al. 2013, 2014a, b). However the source of arsenic, mode of entry and role in the pathogenesis is not established yet.

Agrochemicals are chemical fertilizers and pesticides. Herbicides, insecticides and fungicides are the major categories of pesticides. Urea, phosphate and potash are the main groups of chemical fertilizers used in Sri Lanka (Ekanayake 2009). Chemical fertilizers contain trace amounts of heavy metals and metalloids (Chandrajith et al. 2009). Continuous application of contaminated fertilizers during the past fifty years (since green revolution) may have contributed to increase heavy metals and metalloids in the soil and groundwater aquifers (Weggler et al. 2004). Sri Lanka is the leader in fertilizer usage in the South Asia (Sri Lanka 230.8, Pakistan 217.1, Bangladesh 184.4, India 178.9, Nepal 23.2, kg per hectare respectively of arable land in 2010) (World Bank 2010).

The newly improved varieties of rice cultivated by farmers requires a large amount of urea, triple super phosphate (TSP) and muriate of potash (MOP or potassium chloride), the three main fertilizer varieties subsidized by the government (Ekanayake 2009). WHO classifies pesticides with arsenic as highly hazardous (WHO 2004). Arsenic containing pesticides have been banned in Sri Lanka since 1995 (Fernando and De Silva 2006). Using arsenic to augment potency of agrochemicals is a potential way to increase sales.

**Figure 1:**
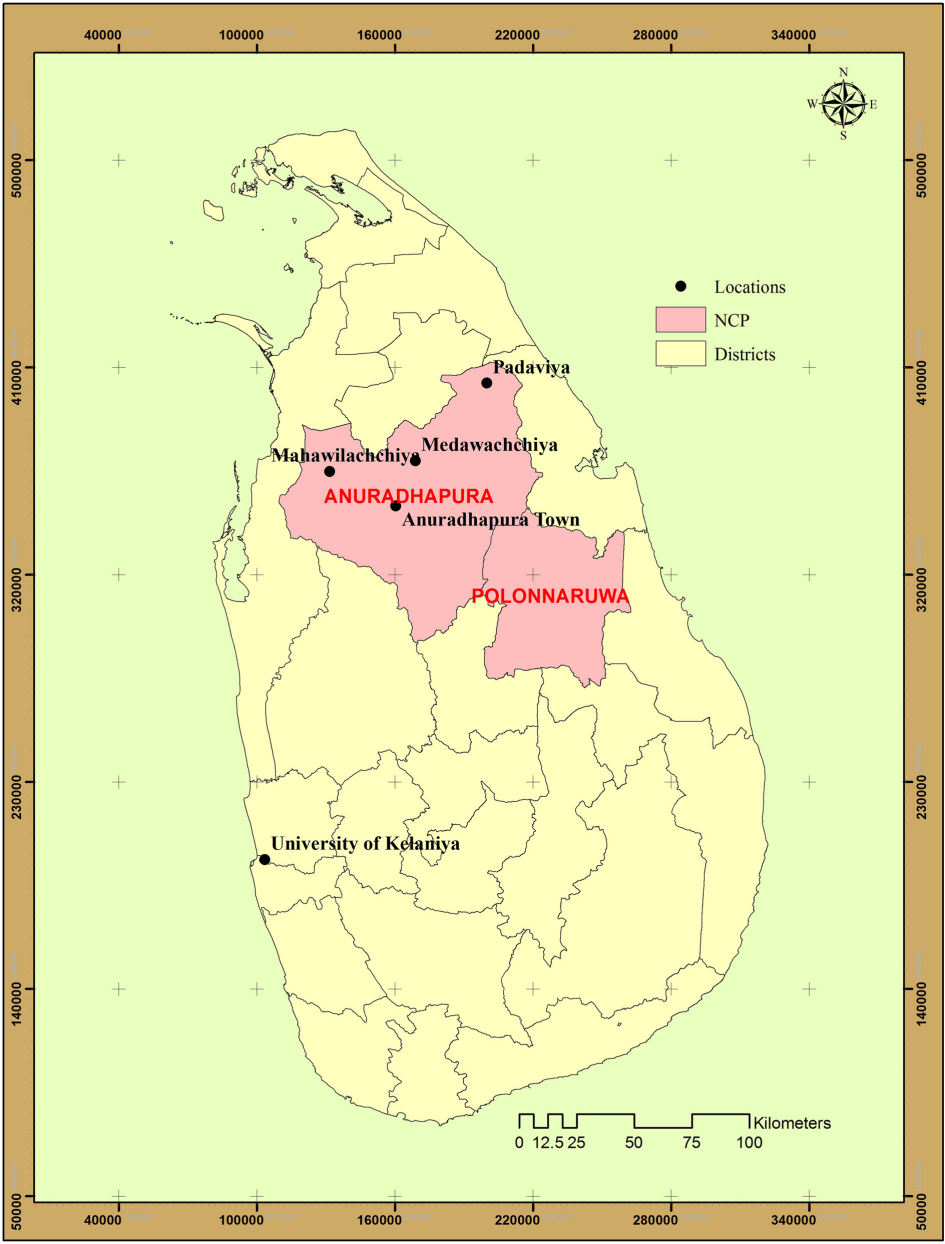
**Sample collection sites in the Anuradhapura district of NCP.**

The objective of the present study was to investigate levels of arsenic contamination in the fertilizer and pesticides used widely for rice cultivation, in four areas of NCP, Padaviya, Medawachchiya, Mahawilachchiya and Anuradhapura town (Figure 1).

## Methods

### Collection of agrochemicals

Samples were collected from four areas in the NCP, Padaviya, Medawachchiya, Mahawilachchiya and Anuradhapura. First three towns are situated in the endemic area and Anuradhapura is the capital of NCP.

Two sets of samples from each type of fertilizer were collected from farmers living close to the above-mentioned four areas. The fertilizer samples were collected from farmers into new polythene bags and sealed. These fertilizers are usually stored in woven polypropylene or polythene bags. (the weight of the fertilizer in a bag or the volume of liquid in a container indicated within parentheses). Samples of urea (50 kg), TSP (50 kg), MOP (50 kg), Eppawala rock phosphate (50 kg), NPK mixture (5 or 10 kg), liquid fertilizer (4 L can), dolomite (50 kg), compost (5 kg), chicken manure (5 kg), cattle manure mix (5 kg), wood charcoal (5 kg), paddy husk charcoal (5 kg) and coir dust (5 kg) were collected from farmers. 238 fertilizer samples were collected. Twelve samples, four each of urea, MOP and TSP were sent to the Institute for Integrated Research in Materials, Environments and Society (IIRMES) laboratory, California State University, Long Beach (CSULB), USA for further analysis. All the others samples were analyzed using atomic absorption spectrometry (AAS) and stored at the department of chemistry, University of Kelaniya.

273 samples of pesticides representing 21 chemical groups (31 brands) were purchased in duplicate, in sealed containers, from vendors in Medawachchiya, Anuradhapura town, Padaviya and Mahawilachchiya. One set was analyzed using AAS and the other set was stored with evidence of proof of purchase at the department of chemistry, University of Kelaniya.

### Treatment of samples

Each sample (0.1 g) was weighed into a 100 ml conical flask, and digested using US EPA method 3050B as described below (United States, Environmental Protection Agency 1996). Each sample was mixed thoroughly to achieve homogeneity and sieved using a USS #10 sieve. All equipment used for homogenization was cleaned thoroughly to minimize the potential cross-contamination. For each digestion procedure, a sample was weighed to the nearest 0.01 g and a 0.1 g sample (dry weight) was transferred to a digestion vessel. Concentrated HNO_3_ (10 ml) was added into it. The sample was heated to 95 ± 5°C and refluxed for 10 to 15 minutes without boiling. The sample was allowed to cool, and then 5 ml of Concentrated HNO_3_ was added and refluxed for 30 minutes. If brown fumes were generated, indicating oxidation of the sample by HNO_3_, this step was repeated (addition of 5 ml of conc. HNO_3_) over and over until no brown fumes were given off by the sample indicating the complete reaction with HNO_3_. The solution was allowed to evaporate to approximately 5 ml without boiling and heated at 95 ± 5°C for two hours. The sample was cooled to room temperature and 2 ml of water and 3 ml of 30% H_2_O_2_ were added. The vessel was covered with a watch glass (care was taken to ensure that losses did not occur due to excessively vigorous effervescence). The sample was heated until effervescence subsides and the vessel was cooled. The adding of 30% H_2_O_2_ in 1 ml aliquots with warming was continued until the effervescence was minimal. The reaction mixture was poured into a 100 ml volumetric flask and diluted to 100 ml. Each fertilizer was digested in duplicates. Spiked samples (10 μg/L) in duplicates were also digested to confirm the methodology. The same procedure repeated without the fertilizer was considered as the control.

### AAS (atomic absorption spectrometry) analysis

GBC 932 plus AAS (GBC scientific equipments, VIC, Australia) equipped with a hydride generation system (GBC 3000) and graphite furnace (GF 3000) with the deuterium background corrector was used to detect arsenic.

All chemicals used were of analytical grade, and the standard solution of arsenic was sourced from Reagecon, Ireland. Chemicals used were concentrated nitric acid (70% AR) (Techno Pharmachem Pvt. Ltd.), HCl (37% AR) (Sigma Aldrich Pvt. Ltd), and high purity hydrogen peroxide (35.5%) (Sigma Aldrich Pvt. Ltd). Laboratory glassware was kept overnight in 10% (v/v) nitric acid.

Standard solutions were prepared daily for analysis. The calibration curves were established with the standard solutions and the square of correlation coefficient (R^2^) was in the range of 0.987 - 0.999 in most occasions while AAS was in use.

### ICP-MS analysis

Major and trace elements were measured using an Inductively Coupled Plasma Mass Spectrometer (ICP-MS; HP 4500, Agilent Technologies, Palo Alto, CA) equipped with a quadrupole analyzer and octopole collision/reaction cell that can be pressurized with either a hydrogen or helium reaction gas. Analysis was done in accordance with United States Environment Protection Agency standards No.6020 M. Samples were injected at the rate of 0.4 mL/min using a peristaltic pump. Carrier Argon gas at a rate of 1.2 L/min was inserted into a Peltier-cooled double-pass spray-chamber through a Babbington-style nebulizer at 2°C. Auxiliary argon gas at the rate of 1.0 L/min and plasma argon at the rate of 12.0 L/min were added for a total of 14.2 L/min separated from nickel cones. The ICP-MS was tuned according to the manufacturer suggested standard settings by running solution of 10 μg/L of Li, Y, Ce, Tl, and Co (Agilent internal standard mix) for resolution and sensitivity. Optimizing plasma conditions to produce low oxide reduced interference levels and doubly charged ions (formation ratio of <1.0% at the plasma conditions of this.) and residual matrix interferences were removed using the collision/reaction processes in the octopole reaction system. Accuracy was measured using the spiked standard solutions. Ultrapure water (MilliQ 18.8 ohms) was used as blank (1 blank per each 10 sample batch).

### Quality control

Precision (reproducibility) was ascertained using within-day replicate analysis of samples. The Relative Standard Deviation (% RSD = SD/χ of the replicate values X 100%; χ is mean value) was calculated to give an indication of sample preparation and analytical precision. Replicates of samples provided an indication of within-day precision. The analytical detection limit was calculated as the concentration of the element which gave a detectable signal above the background noise at greater than the 99% confidence level, and the detection limit was calculated as the mean of blanks plus 3 times the standard deviation of the mean.

## Results and discussion

NCP encompasses 16% (10472 km^2^) of the landmass in Sri Lanka and is inhabited by 6.2% (1,259,421) of its population (Department of Census, Sri Lanka 2012). According to the Ministry of Health 73% (50382) of CKDu patients live in NCP (Ministry of Health 2014).

Results of 226 fertilizer samples analyzed at Kelaniya University using AAS are shown in Table 1. Arsenic content of the fertilizers (Table 2) tested in IIRMES laboratory, CSULB, USA was similar to the results obtained from the University of Kelaniya, Sri Lanka. The analysis demonstrated TSP as a rich source of other heavy metals as well (Table 2). The highest arsenic content was seen in TSP and the next highest was in rock phosphate (3Ca(PO_4_)_2_ F.Cl.OH^−^) produced at Eppawala (a mining area in the NCP) and in dolomite (CaCO_3_ MgCO_3_) produced in Naula area in the Central province of Sri Lanka.

**Table 1:** **Arsenic content of the synthetic and natural fertilizers available in Sri Lanka**

**Type of fertilizer**	**No of samples**	**Range of as content (mg/kg)**	**Mean as content (mg/kg)**	**Imported quantity in 2012 (MT)**
TSP*^+^	17	25.49-37.86	31.00	108229
Eppawala rock phosphate	15	3.4-21.81	8.56	PP
Dolamite	15	6.01-7.61	6.58	PP
NPK mixture#	15	1.95-7.28	5.88	4879
Urea*^+^	18	0.88-1.09	0.92	302831
Ammonium sulphate*	10	0.71-1.21	0.94	77199
Cattle manure mix	18	0.76-1.02	0.84	PP
MOP*^+^	18	ND-1.02	0.44	111855
Compost	20	ND-1.34	0.41	PP
Chicken manure	18	0.25-0.72	0.38	PP
Liquid fertilizer^#^	15	ND-0.65	0.33	241222
Paddy husk charcoal	16	ND-0.23	0.10	PP
Coir dust	16	ND	0.00	PP
Wood charcoal	15	ND	0.00	PP

PP Purchased and produced in Sri Lanka. *The government imports and heavily subsidizes these fertilizers. ^+^mainly used in rice cultivation ^#^Imported by private sector and not used in paddy cultivation.

**Table 2:** **Trace metal profiles of commonly used fertilizers in Sri Lanka**

**(mg/kg)**	**Urea**		**MOP**		**TSP**	
**Element**	**Mean**	**Range**	**Mean**	**Range**	**Mean**	**Range**
Al	2.6	1.0-3.3	151.3	97.9-231.1	9939.0	8923.0-11290.0
Sb	0.1	0.1-0.2	0.1	0.1-0.1	6.0	5.7-6.0
As	0.1	ND-0.3	0.3	0.2-0.4	28.9	26.5-31.9
Ba	0.1	0.1-0.1	1.1	1.0-1.3	79.1	77.6-83.4
Be	0.1	0.1-0.1	0.1	0.1-0.2	2.2	2.1-2.3
Cd	ND	ND	0.1	0.1-0.2	2.0	1.9-2.0
Cr	ND	ND	1.2	0.4-2.7	29.3	22.6-33.7
Co	0.1	0.1-0.1	0.2	0.2-0.3	6.0	5.9-6.4
Cu	0.2	0.1-0.4	0.3	0.3-0.4	15.0	14.2-16.0
Fe	1.0	ND-1.7	2371.3	2252.3-2634.3	11215.3	10910.3-11760.3
Pb	0.2	0.2 - 0.2	0.8	0.8-0.9	252.5	251.7-263.9
Mn	0.3	0.1-0.4	12.3	11.6-13.7	1948.0	1886.0-2034.0
Ni	1.0	0.2-3.7	0.3	0.2-0.5	25.0	23.9-27.1
Se	0.2	ND-0.5	1.7	1.4-2.1	2.0	1.2-2.5
Ag	0.1	0.1-0.1	0.1	0.1-0.1	0.3	0.1-0.3
Sr	0.1	ND-0.1	10.2	9.8-10.6	245	230-277.9
Tl	0.1	ND-0.1	0.1	ND-0.2	0.5	0.5-0.5
Sn	0.1	0.1-0.2	0.2	0.2-0.3	0.7	0.7-0.7
Ti	ND	ND	4.1	2.6-5.0	439.6	379.0-496.3
V	0.2	0.2-0.4	0.3	0.2-0.5	37.1	34.9-39.3
Zn	ND	ND	0.8	0.2-1.3	489.8	443.6-544.0

Natural (or organic) fertilizers such as cattle manure, compost, chicken manure, paddy husk, coir, wood charcoal all contained very low amounts of arsenic. Cattle manure may be contaminated with arsenic from grass grown with TSP and chickens may be fed with arsenic containing Roxarsone, a food additive and an anti-protozoan drug. The government does not subsidize for pesticides. Despite the fact that the import of arsenic containing pesticides is illegal, all 31 pesticide brands belonging to 21 active ingredients contained arsenic (Table 3).

**Table 3:** **Arsenic content of the pesticides available in Sri Lanka**

**Active ingredient**	**Type**	**No of samples**	**Range of as content (μg/kg)**	**Mean as content (μg/Kg)**	**Imported quantity in 2012 (MT)**
Dimethoate	I	12	965-2457	1957	NA
Glyphosate	H	18	858-2586	1896	5295
Fenoxaprop-p-ethyl	H	12	1254-2578	1835	41
Mancozeb	F	15	458-2478	1680	692
Carbofuran	I	18	831-2458	1578	299
Propanil	H	12	512-2584	1324	1094
Methomyl	I	12	1112-1458	1279	09
Quinalphos	I	12	928-1893	1278	08
Carbendazim	F	12	1163-1458	1278	20
Profenofos	I	12	458-1452	968	141
MCPA	H	18	458-1496	967	686
Bispyribac Na	H	12	721-1458	923	50
Methoxyfenozide	I	12	872-911	902	04
Thiamethoxam	I	12	542-1024	874	08
Chlophyriphos	I	18	654-1365	804	420
Phenthoate	I	12	565-1258	785	32
Diazinon	I	12	625-995	708	197
Oxyfluorfen	H	06	423-788	602	33
Pretilachlor + Pyribenzoxim	H	12	415-655	530	102
Tebuconazole	F	12	288-680	420	19
Imidacloprid	I	12	180-359	239	33

F = fungicide H = herbicide I = insecticide.

TSP is one of the major sources of arsenic in disease endemic areas. The quantity of arsenic in TSP is 15 times more than that insecticide Dimethoate which contained the highest amount of arsenic among the pesticides. In 2012, the total amount of TSP imported to Sri Lanka was 1.08×10^6^ kg amounting to 2100 kg of arsenic. The total amount of arsenic in pesticides imported in 2012 do not exceed 15 kg (mean arsenic content of each pesticide was multiplied by the total imported). Thus, confirming fertilizers (TSP and others) as the major source of arsenic. There is documented evidence since 1968 that Sri Lanka has been importing phosphate fertilizers, and its use has increased during the past 40 years. Although no regulations are currently in existence about fertilizer contaminated with arsenic, Government Gazette Notification No. 1190/24 of 29th June 2001 under the Control of Pesticides Act No. 33 of 1980 banned arsenic as an active ingredient in pesticides in Sri Lanka (Fernando and De Silva 2006). Level of trace metals in the fertilizers including arsenic can vary widely according to the country of origin, but determining the country of origin of the fertilizers is extremely difficult (Dissanayake and Chandrajith 2009). Arsenic content in the phosphate fertilizers available to the Sri Lankan farmers is comparatively high when compared with the arsenic content in phosphate fertilizers available in other countries (Mortvedt 1996).

Imported granular TSP is mainly used for perennial crops such as rice and vegetables. Eppawala rock phosphate (from an open-pit mine in NCP of Sri Lanka) has a very low solubility and is only used in long-term crops such as tea, rubber and coconut mainly in the wet zone of Sri Lanka (Dissanayake and Chandrajith 2009). Its usage in the CKDu endemic areas is minimal. Apatite structure in phosphate rock includes heavy metals such as As, Cd, Cr, Hg, Pb, Se, U and V. Aside from its presence in the phosphate rock, arsenic may be introduced as an impurity in the sulfuric and phosphoric acids used in the manufacturing process of superphosphate. Sulfuric acid manufactured from pyrite is used extensively in the fertilizer industry; and often contains considerable amount of arsenic (Tremearne and Jacob 1941). Phosphoric acid manufactured through sulfuric acid process and from rock phosphate also contains arsenic as an impurity.

In the disease endemic region arsenic content in soil gradually decreases as we descend from the surface downwards of the earth’s crust, implying that it is not present naturally but has been introduced most probably due to anthropogenic activity (Fonseka et al. 2012). The soil is an important sink for arsenic compounds but the amount retained depends on the nature of the soil type. Arsenic and phosphorous are both group V elements with similar properties. However phosphorous is essential for plants but arsenic is toxic to both plants and animals. Since both have similar chemical properties arsenate and phosphate compete for same sorption sites in the root, resulting in reduction in sorption and increase in the arsenic concentration in ground water in a phosphate rich environment (Smith and Naidu 2009). Phosphorus containing fertilizer added to soil increases the mobility of arsenic and consequently, arsenic becomes more biologically available (Davenport and Peryea 1991).

Arsenic in water can exist both as organic and inorganic forms. However inorganic arsenic is the primary form that poses human health risk and exists as arsenate (AsO_4_^3−^) (+5) or arsenite (AsO_3_^3−^) (+3). In oxidizing conditions (typically aerobic), arsenate dominates, and in reducing conditions (typically anaerobic and anoxic, such as flooded rice fields), arsenite (+3) dominates. Arsenite is more soluble than arsenate at neutral pH and at ion concentrations typical of fresh water (Zhao et al. 2010). Chemical interactions among arsenates, phosphates and carbonates are important to understand their ability of adsorption to soil, as sorption properties of these anions are almost identical. The arsenites (+3) are more soluble, mobile and toxic than the arsenates but both forms have harmful effects on humans, plants and animals. The World Health Organization (WHO) has recommended maximum permissible level for arsenic in drinking water as 10 μg/L (World Health Organization 2011).

Arsenic in soil can contaminate drinking water and food crops including vegetables, fruits and grain. Accumulation of heavy metals in vegetables occurs after application of phosphate fertilizers (Jiao et al. 2012). Recently, Codex has adopted a maximum level for inorganic arsenic in polished rice as 200 μg/kg (Food and agricultural organization of the United Nations 2014). Chronic arsenic poisoning causes many health hazards and entry of minute quantities of arsenic into the human body through the food chain over several years can cause many non-communicable diseases (Kapaj et al. 2006; Golka et al. 2010). Increased amounts of arsenic in urine have been shown in two previous studies among people living in CKDu endemic areas (Jayasumana et al. 2013; Jayatilake et al. 2013). Other than arsenic Cr, Ni, Pb and V content in phosphate fertilizers is also high. Nephrotoxicity of these heavy metals on animals have been discussed widely (Vyskocil et al. 1994; Loghman 1997; de la Torre et al. 1999; Sahu et al. 2014). However, a possible role for Cr, Ni, Pb and V in CKDu in Sri Lanka is not studied in-depth. The hypothesized interactions of these metals in the presence of high levels of arsenic particularly when present in drinking water from shallow wells with a high level of hardness and the role of these metal complexes in the causation of CKDu has been discussed in a previous publication by the current authors (Jayasumana et al. 2013).

One of the major limitations of the study is the absence of chain of custody in the collection of agrochemicals. However receipts of all pesticides purchased and few photographs of fertilizer samples being taken from farmers are available. We could only repeat the analysis in ICP-MS in selected fertilizer samples (5.3%) due to resource limitations.

CKD epidemic in Central America with similar histological findings (López-Marín et al. 2014) and it’s association with large-scale cultivation of sugar cane (Orantes et al. 2014) have shown remarkable similarities with the CKDu epidemic in Sri Lanka. Recent research has shown increased amount of arsenic in water and soil in the endemic areas in El Salvador (Lopez et al. 2013).

## Conclusion

Findings suggest that agrochemicals especially phosphate fertilizers are a major source of inorganic arsenic in CKDu endemic areas in Sri Lanka. Study highlights the magnitude of an environmental issue that has received little attention. Increased arsenic contamination of the soil and ground water can adulterate food and drinking water. Arsenic content in the organic fertilizer available in Sri Lanka is comparatively low and hence the farmers should be encouraged to minimize the use of imported chemical fertilizer and use organic fertilizers in order to avoid further environmental damage and human health hazards.

## Abbreviations

AAS: Atomic absorption spectrometry
CKDu: Chronic kidney disease of unknown etiology
CSULB: California state university, long beach
ICP-MS: Inductively coupled plasma mass spectrometer
IIRMES: Integrated research in materials, environments and society
MoH: Ministry of health
MOP: Muriate of potash
NCP: North central province
TSP: Triple super phosphate

## References

[CR1] Athuraliya NTC, Abeysekera TDJ, Amerasinghe PH, Kumarasiri R, Bandara P, Karunaratne U, Milton AH, Jones AL (2011). Uncertain etiologies of proteinuric-chronic kidney disease in rural Sri Lanka. Kidney Int.

[CR2] Bandara JMRS, Wijewardena HVP, Liyanege J, Upul MA, Bandara JMU (2010). Chronic renal failure in Sri Lanka caused by elevated dietary cadmium: Trojan horse of the green revolution. Toxicol Lett.

[CR3] Chandrajith R, Seneviratna S, Wickramaarachchi K, Attanayake T, Aturaliya TNC, Dissanayake CB (2009). Natural radionuclides and trace elements in rice field soils in relation to fertilizer application: study of a chronic kidney disease area in Sri Lanka. Environ Earth Sci.

[CR4] Davenport JR, Peryea FJ (1991). Phosphate fertilizers influence leaching of lead and arsenic in a soil contaminated with lead arsenate. Water Air Soil Pollut.

[CR5] de la Torre A, Granero S, Mayayo E, Corbella J, Domingo JL (1999). Effect of age on vanadium nephrotoxicity in rats. Toxicol Lett.

[CR6] Department of Census, Sri Lanka (2012), Results of census of population and housing Sri Lanka. http://www.statistics.gov.lk/PopHouSat/CPH2011/index.php?fileName=CPH%202011_R1&gp=Activities&tpl=3 Accessed on 21 Nov 2014

[CR7] Dissanayake CB, Chandrajith R (2009) Phosphate mineral fertilizers, trace metals and human health J Natn Sci Foundation Sri Lanka 37:153–165.

[CR8] Ekanayake H (2009). The Impact of Fertilizer Subsidy on Paddy Cultivation in Sri Lanka. Staff Studies.

[CR9] Fernando R, De Silva S (2006). Banned chemicals in Sri Lanka.

[CR10] Fonseka SI, Amarasinghe MD, Paranagama PA (2012). Preliminary investigations on presence of arsenic in Sri Lankan soils and plants.

[CR11] Food and agricultural organization of the United Nations (2014), Arsenic in rice. [http://www.fao.org/news/story/en/item/238802/icode/] Accessed 21 Nov 2014

[CR12] Golka K, Hengstler JG, Marchan R, Bolt HM (2010). Severe arsenic poisoning: One of the largest man-made catastrophies. Arch Toxicol.

[CR13] Jayasumana MACS, Paranagama PA, Amarasinghe MD, Wijewardane KMRC, Dahanayake KS, Fonseka SI, Rajakaruna KDLMP, Mahamithawa AMP, Samarasinghe UD, Senanayake VK (2013). Possible link of Chronic arsenic toxicity with Chronic Kidney Disease of unknown etiology in Sri Lanka. J Nat Sci Res.

[CR14] Jayasumana C, Gajanayake R, Siribaddana S (2014). Importance of Arsenic and pesticides in epidemic chronic kidney disease in Sri Lanka. BMC Nephrol.

[CR15] Jayasumana C, Gunatilake S, Senanayake P (2014). Glyphosate, hard water and nephrotoxic metals: Are they the culprits behind the epidemic of chronic kidney disease of unknown etiology in Sri Lanka?. Int J Environ Res Public Health.

[CR16] Jayatilake N, Mendis S, Maheepala P, Mehta FR (2013). Chronic kidney disease of uncertain aetiology: prevalence and causative factors in a developing country. BMC Nephrol.

[CR17] Jiao W, Chen W, Chang AC, Page AL (2012). Environmental risks of trace elements associated with long-term phosphate fertilizers applications: A review. Environ Pollut.

[CR18] Kapaj S, Peterson H, Liber K, Bhattacharya P (2006). Human health effects from chronic arsenic poisoning-a review. J Environ Sci Health A Tox Hazard Subst Environ Eng.

[CR19] Loghman-Adham M (1997). Renal effects of environmental and occupational lead exposure. Environ Health Perspect.

[CR20] Lopez DL, Ribo A, Quinteros E, Mejia R, Lopez A, Jovel R, Vandervort D, Orantes C (2013). Heavy metals, arsenic, and pesticide contamination in an area with high incidence of chronic kidney disease of non-traditional causes in El Salvador.

[CR21] López-Marín L, Chávez Y, García XA, Flores WM, García YM, Herrera R, Almaguer M, Orantes CM, Calero D, Bayarre HD, Amaya JC, Magaña S, Espinoza PA, Serpas L (2014). Histopathology of chronic kidney disease of unknown etiology in Salvadoran agricultural communities. MEDICC Rev.

[CR22] Ministry of Health (2009). Chronic Kidney Disease of Unknown Etiology, Circular no 01-10/2009.

[CR23] Ministry of Health (2014), Data presented at the presidential task force for prevention of kidney diseases, Colombo, Sri Lanka. 6th June

[CR24] Mortvedt JJ (1996). Heavy metal contaminants in inorganic and organic fertilizers. Fertil Res.

[CR25] Nanayakkara S, Komiya T, Ratnatunga N, Senevirathna STMLD, Harada KH, Hitomi T, Gobe G, Muso E, Abeysekera T, Koizumi A (2012). Tubulointerstitial damage as the major pathological lesion in endemic chronic kidney disease among farmers in North Central Province of Sri Lanka. Environ Health Prev Med.

[CR26] Nanayakkara S, Senevirathna STMLD, Karunaratne U, Chandrajith R, Harada KH, Hitomi T, Watanabe T, Abeysekera T, Aturaliya TNC, Koizumi A (2012). Evidence of tubular damage in the very early stage of chronic kidney disease of uncertain etiology in the North Central Province of Sri Lanka: a cross-sectional study. Environ Health Prev Med.

[CR27] Orantes CM, Herrera R, Almaguer M, Brizuela EG, Núñez L, Alvarado NP, Fuentes EJ, Bayarre HD, Amaya JC, Calero DJ, Vela XF, Zelaya SM, Granados DV, Orellana P (2014). Epidemiology of chronic kidney disease in adults of Salvadoran agricultural communities. MEDICC Rev.

[CR28] Peiris-John RJ, Wanigasuriya JK, Wickremasinghe AR, Dissanayake WP, Hittarage (2006). Exposure to acetylcholinesterase-inhibiting pesticides and chronic renal failure. Ceylon Med J.

[CR29] Sahu BD, Koneru M, Bijargi SR, Kota A, Sistla R (2014). Chromium-induced nephrotoxicity and ameliorative effect of carvedilol in rats: Involvement of oxidative stress, apoptosis and inflammation. Chem Biol Interact.

[CR30] Smith E, Naidu R (2009). Chemistry of inorganic arsenic in soils: Kinetics of arsenic adsorption - Desorption. Environ Geochem Health.

[CR31] Tremearne TH, Jacob KD (1941). Arsenic in natural phosphates and phosphate fertilizers. United States Department Agric Tech Bullet.

[CR32] United States, Environmental Protection Agency (1996), Acid digestion method 3050B http://www.epa.gov/osw/hazard/testmethods/sw846/pdfs/3050b.pdf Accessed 5 Feb 2014

[CR33] Vyskocil A, Viau C, Cízková M (1994). Chronic nephrotoxicity of soluble nickel in rats. Hum Exp Toxicol.

[CR34] Wanigasuriya KP, Peiris-John RJ, Wickremasinghe R (2011). Chronic kidney disease of unknown aetiology in Sri Lanka: is cadmium a likely cause?. BMC Nephrol.

[CR35] Weggler K, McLaughlin MJ, Graham RD (2004). Effect of Chloride in Soil Solution on the Plant Availability of Biosolid-Borne Cadmium. J Environ Qual.

[CR36] WHO (2004). Recommended classification of pesticides by hazard and guidelines to classification.

[CR37] World Bank (2010), Fertilizer consumption data. http://data.worldbank.org/indicator/AG.CON.FERT.ZS Accessed 21 Nov 2014

[CR38] World Health Organization (2011), Arsenic in Drinking water. http://www.who.int/water_sanitation_health/dwq/chemicals/arsenic.pdf Accessed 21 Nov 2014

[CR39] Zhao F-J, McGrath SP, Meharg AA (2010). Arsenic as a food chain contaminant: mechanisms of plant uptake and metabolism and mitigation strategies. Annu Rev Plant Biol.

